# Demographic Tendencies and Hospitalization Outcomes Among Inpatient Admissions of Osteoarthritis in the Midwest: A 2016 State Inpatient Database Study

**DOI:** 10.7759/cureus.7959

**Published:** 2020-05-04

**Authors:** Charles A Gusho, Mark Jenson

**Affiliations:** 1 Orthopaedic Surgery, Medical College of Wisconsin - Green Bay, De Pere, USA; 2 Family Medicine, Medical College of Wisconsin - Green Bay, De Pere, USA

**Keywords:** osteoarthritis, state inpatient database

## Abstract

Objective

To assess the inpatient prevalence of osteoarthritis in a Midwestern state and to identify trends in demographics and hospital outcomes.

Methods

The Wisconsin State Inpatient Sample Database (2016) was queried to identify hospitalization records with a primary diagnosis of osteoarthritis. Bivariate correlation, descriptive statistics, and single-layer mean comparison were used for categorical and continuous data within the International Classification of Diseases, Tenth Revision, Clinical Modification (ICD-10-CM) sub-groups.

Results

In 2016, there were 64,805 admissions of osteoarthritis. The most common (>0.09%) were the right knee osteoarthritis (24.5%), left knee osteoarthritis (23%), right hip osteoarthritis (16.9%), left hip osteoarthritis (14.3%), knee unspecified osteoarthritis (11.5%), bilateral knees osteoarthritis (7%), and right shoulder osteoarthritis (2%). The mean age on admission was 67 years for each hip osteoarthritis, 66 years for each knee osteoarthritis, and 69 years for right shoulder osteoarthritis. The mean length of stay was 3.15 days for bilateral knee osteoarthritis and 1.92 days for the right shoulder osteoarthritis. Total inpatient charges and in-hospital mortality were highest in right shoulder osteoarthritis (USD 52,699.40 [0.6%]; N = 6), and total charges were lowest in right and left hip osteoarthritis (44,689.54 and 44,427.33, respectively). A greater frequency of females and Caucasians was consistently admitted within each of the included ICD-10-CM OA sub-groupings. Age was correlated with charge in the left hip osteoarthritis (r = 0.050) and right shoulder osteoarthritis (r = 0.068), and was negatively correlated with charge in the bilateral knee osteoarthritis (r = -0.115), right knee osteoarthritis (r = -0.054), and left knee osteoarthritis (r = -0.060).

Conclusions

In Wisconsin, with somewhat of a generalizability to other Midwestern states, attention should be given to Caucasian, elderly, and female patients with osteoarthritis of the hip and knee. Further studies are needed to broaden the understanding of cost utilization, how charges and hospital stay compare nationwide, and where preventative efforts are needed.

## Introduction

Osteoarthritis (OA) is considered the most common disorder of joints, affecting approximately 27 million Americans every year [1]. OA is typically managed non-surgically, with treatments that include low-dose steroids, non-steroidal anti-inflammatory drugs, and, despite its consensus recommendation, hyaluronic acid, bone marrow stem cells, and platelet-rich plasma. However, if presenting symptoms are severe or if a radiographic disease is advanced, surgical management such as joint arthroplasty (replacement) is the treatment of choice. Risk factors for OA are grouped into non-modifiable risk factors, such as age, sex, race and ethnicity, and modifiable risk factors such as diet, occupation, and obesity. Caucasian, post-menopausal, elderly females are at a greater risk of developing OA, as well as individuals with a high body mass index (BMI) due to increased forces of weight-bearing causing mechanical joint destruction [2]. Presently, the incidence of OA in Americans is increasing along with the aging population, and, consequently, the volume of total joint arthroplasty in the United States is expected to sustain a near 71% and 85% increase in hip and knee replacements over the next decade, respectively [3].

The predicted increase in volume suggests a nationwide surge in healthcare expenditure through joint arthroplasty and inpatient hospital stay, though its rise will be amplified in states with a higher proportion (>30%) of obese and non-Hispanic white (NHW) adult residents, such as Wisconsin [4]. Therefore, states including Wisconsin may benefit from prevalence and cost-utilization studies focused on the demographics and inpatient economic burden of OA, which may motivate preventative efforts or targeted inpatient care of individuals at risk.

In Wisconsin, however, reliable statewide data to assess the inpatient burden of OA is missing. This study is the first of its kind and explores the prevalence of the most common types of osteoarthritic admissions. It highlights the key differences in hospital outcomes among each of the most common groups, such as length of stay, mortality, disposition, and total charges during hospitalization, as well as various demographic trends, using Wisconsin’s largest state inpatient database dispensed by the Agency for Healthcare Research and Quality (AHRQ).

## Materials and methods

Database source

A retrospective analysis was conducted using the Healthcare Cost and Utilization Project’s (HCUP) Statewide Inpatient Database (SID) for Wisconsin in the year 2016 [5]. The 2016 HCUP-SID from Wisconsin consists of inpatient admissions from 153 acute-care, non-federal community hospitals across the state. Around 600,000 records of inpatient discharges are documented from these Wisconsin hospitals annually, with an additional 28,000 coming from excluded hospitals such as non-community hospitals, rehabilitation hospitals, and hospitals not included in the HCUP survey. The inpatient data from 2016 represents a recent (less than five years old) homogenous dataset. Additionally, in using a single database corresponding to a single U.S. state, discharge weighting criteria were not necessary as this was not a pooled nationwide sample.

Inclusion criteria

The study included adult patients above 18 years of age with a primary diagnosis of OA using the International Classification of Diseases, Tenth Revision, Clinical Modification (ICD-10-CM) diagnosis codes. Then, based on the ICD-10-CM diagnosis codes, patients were further stratified into ICD-10-CM subsets within the OA musculoskeletal (MUS006) cluster. In the SID database, each of the ICD-10-CM diagnosis codes is further classified into clinically meaningful categories by the AHRQ’s Clinical Classification Software (CCS), and for the purposes of this study, the focus was on the “MUS006” cluster, which represents a portion of the “musculoskeletal” group containing all of the admitting diagnoses of OA [6]. Using this sub-grouping of the SID, a large population of relatively similar conditions within a single cohort was identified. Further divisions (represented by “M”) within this cluster include primary generalized OA (ICD-10-CM M150), erosive OA (M154), poly-osteoarthritis (M159), OA of the hip (M160, M1610-12, M162, M1631-2, M1651-2, M166-7, M169), knee (M170, M1710-12, M172, M1731-2, M174-5, M179), hand and wrist (M1811, M19031, 19042), shoulder (M19011-2, M19019, M19111-2, M19119, M19211-2), elbow (M19021-2, M19121), and foot and ankle (M19071-2, M19171-2), and unspecified OA (M1990), primary OA, unspecified site (M1991), and post-traumatic OA, unspecified site (M1992). For joints such as the knee, hip, shoulder, elbow, and foot and ankle, ICD-10-CM codes are further distinguished by laterality, primary versus secondary OA, post-traumatic versus atraumatic OA, and those unspecified.

Variables of interest

The first goal was to identify differences in the demographic trends among each of the most common ICD-10-CM admitting diagnoses within the OA group. These variables included age, sex, and race. The second goal was to study the hospital outcome variables including length of stay, total inpatient hospital charges, hospital disposition using Centers for Medicare and Medicaid Services (CMS) coding, and inpatient mortality [7]. This second goal was accomplished by calculating the inpatient length of stay as the number of days spent within the hospital as opposed to nights, and the total inpatient charges per admission were cleaned data in U.S. dollars (USD) devoid of non-covered charges and professional fees [8].

Analytic approach

Data splitting was used to group demographic variables and hospital outcome variables by ICD-10-CM admitting diagnosis codes. The prevalence of OA in Wisconsin admissions from the year 2016 was calculated using descriptive statistics, and only subset ICD-10-CM codes with significant sample sizes (>0.09% of total cumulative admissions in 2016) were included for subsequent analyses. The mean and standard deviation (SD) were used to describe continuous variables of interest (age, total charges, length of stay, in-hospital mortality) as well as categorical variables such as sex, race, and hospital disposition, each of which was then decrypted using numerical codes. Independent sample t-tests were used to compare mean total hospital charges, age, and lengths of hospital stay, and the chi-square test was used to compare in-hospital mortality between groups with hospital death. Single-layer comparison of means was used to analyze age-specific differences in total charges and length of stay. Pearson’s bivariate test was used to assess the correlative relationship between age (in years) on admission and total charges (in USD), using 55 years as an arbitrary cut-off. All statistical analyses in this study were performed using SPSS Version 26 (IBM Corp., Armonk, NY, USA) [9].

Ethical approval

Diagnostic and procedural information in the SID was identified using the ICD-10-CM and CCS cluster codes. To protect the privacy of physicians, hospitals, and patients, the entire dataset was de-identified using patient key identifiers (noted in the dataset as KEY_ID). The use of administrative state inpatient databases under the HCUP, according to the AHRQ of the U.S. Department of Health and Human Services, does not require approval from an Institutional Review Board as the SID is a publicly available, de-identified inpatient dataset.

## Results

Characteristics

This study analyzed 64,805 admitting ICD-10-CM diagnoses of OA presenting to 153 different hospitals in Wisconsin in 2016. Only ICD-10-CM diagnoses representing > 0.09% of total cumulative admissions (N = 2,730,832) were analyzed. The sum of the remaining ICD-10-CM codes within the CCS “MUS006” category (N = 4,721) represents a negligible percentage (<0.09%) of total osteoarthritic admissions and was not included in subsequent analyses. Among the included admissions codes (N = 60,084), unilateral primary OA of the right knee was the most prevalent type (N = 14,771 [24.58%]) followed by unilateral left knee OA (N = 13,860 [23.07%]), unilateral right hip OA (N = 10,198 [16.97%]), unilateral left hip arthritis (N = 8,651 [14.40%]), unspecified OA of the knee (N = 6,956 [11.58%]), bilateral primary OA of the knee (N = 4,237 [7.05%]), and primary OA of the right shoulder (N = 1,411 [2.35%]) (Table 1).

**Table 1:** Prevalence of the seven most common ICD-10-CM admissions within the MUS006 cluster, totaling 60,084 total osteoarthritis admissions in 2016. ICD-10-CM, International Classification of Diseases, Tenth Revision, Clinical Modification; M1611, unilateral primary right hip osteoarthritis; M1612, unilateral primary left hip osteoarthritis; M170, bilateral primary knee osteoarthritis; M1711, unilateral primary right knee osteoarthritis; M1712, unilateral primary left knee osteoarthritis; M179, unspecified knee osteoarthritis; M19011, primary right shoulder osteoarthritis.

ICD-10-CM	M1611	M1612	M170	M1711	M1712	M179	M19011
Frequency	10,198	8,651	4,237	14,771	13,860	6,956	1,411
Weighted %	16.97	14.40	7.05	24.58	23.07	11.58	2.35

Inpatient demographics

Patients admitted for unilateral primary OA of the right shoulder were oldest (mean age ± SD: 69.62 ± 9.507 years) compared with unilateral primary OA of the left hip (67.06 ± 10.732 years; p = 0.02), unilateral primary OA of the right hip (67.05 ± 10.499 years; p = 0.083), unspecified OA of the knee (66.94 ± 9.985 years; p < 0.01), unilateral primary OA of the right knee (66.84 ± 9.7 years; p < 0.01), unilateral primary OA of the left knee (66.65 ± 9.795 years; p < 0.01), and bilateral primary OA of the knees (64.46 ± 9.156 years; p = 0.16). A greater frequency of females and Caucasians was consistently admitted within each of the included ICD-10-CM OA sub-groupings (Table 2).

**Table 2:** Age (in years) at admission, sex, and race analysis among the most common ICD-10-CM osteoarthritis admissions. ICD-10-CM, International Classification of Diseases, Tenth Revision, Clinical Modification; M1611, unilateral primary right hip osteoarthritis; M1612, unilateral primary left hip osteoarthritis; M170, bilateral primary knee osteoarthritis; M1711, unilateral primary right knee osteoarthritis; M1712, unilateral primary left knee osteoarthritis; M179, unspecified knee osteoarthritis; M19011, primary right shoulder osteoarthritis; NA, Native American; SD, standard deviation. *Significant at 0.01 level compared to M19011 (two-tailed, independent sample t-test).

ICD-10-CM	M1611	M1612	M170	M1711	M1712	M179	M19011
Age, mean ± SD	67.05 ± 10.5*	67.06 ± 10.73*	64.46 ± 9.2	66.84 ± 9.7	66.65 ± 9.8	66.94 ± 9.9*	69.62 ± 9.5
Gender, %							
Males	43.0	45.3	42.0	37.3	38.5	37.3	45.7
Females	57.0	54.7	58.0	62.7	61.5	62.7	54.3
Race, %							
White	94.1	95.6	94.7	93.7	93.5	92.7	95.4
Black	3.4	3.3	3.5	3.9	4.2	4.8	3.2
Hispanic	0.8	0.5	1.1	1.7	1.6	1.4	0.9
Asian	0.1	0.3	0.5	0.2	0.3	0.4	0.2
NA	0.3	0.3	0.1	0.4	0.4	0.6	0.2
Other	0.0	0.0	0.1	0	0.0	0.0	0

Hospital outcomes

The mean inpatient length of stay was highest in the bilateral primary knee OA group (3.15 days) compared with unspecified OA of the knee (2.73 days; p < 0.01), primary left hip OA (2.67 days; p = 0.228), primary left knee OA (2.67 days; p < 0.01), primary right knee OA (2.63 days; p = 0.779), primary right hip OA (2.56 days; p < 0.01), and lowest in the primary right shoulder OA (1.92 days; p < 0.01) (Table 3).

**Table 3:** Mean inpatient length of stay, mean inpatient hospital charges, and in-hospital mortality among the most common ICD-10-CM osteoarthritis admissions. ICD-10-CM, International Classification of Diseases, Tenth Revision, Clinical Modification; M1611, unilateral primary right hip osteoarthritis; M1612, unilateral primary left hip osteoarthritis; M170, bilateral primary knee osteoarthritis; M1711, unilateral primary right knee osteoarthritis; M1712, unilateral primary left knee osteoarthritis; M179, unspecified knee osteoarthritis; M19011, primary right shoulder osteoarthritis; USD, U.S. dollars. *Significant at 0.01 level compared to M170 (two-tailed, independent sample t-test). **Significant at 0.01 level compared to M19011 (chi-square test).

ICD-10-CM	M1611	M1612	M170	M1711	M1712	M179	M19011
Mean total charges (USD)	47,451*	47,864*	61,460	44,689*	44,427*	45,799*	52,699*
Mean length of stay (days)	2.56	2.67*	3.15	2.63*	2.67	2.73*	1.92*
In-hospital mortality (frequency, %)	N = 9, 0.1**	N = 0, 0.0	N = 0, 0.0	N = 0, 0.0	N = 0, 0.0	N = 5, 0.1**	N = 9, 0.6

The mean total inpatient charges were highest in bilateral primary OA of the knee (USD 61,460) compared with primary right shoulder OA (USD 52,669; p < 0.01), primary left hip OA (USD 47,864; p < 0.01), primary right hip OA (USD 47,451; p < 0.01), unspecified OA of the knee (USD 45,799; p < 0.01), primary right knee OA (USD 44,689; p < 0.01), and primary left knee OA (USD 44,427; p < 0.01). Inpatient hospital mortality was highest in primary right shoulder OA (N = 9 [0.6%]) when compared with primary right hip OA (N = 9 [0.1%]; p < 0.01) and unspecified OA of the knee (N = 5 [0.1%]; p < 0.01). Each of the remaining sub-groupings had no incident inpatient mortality (Table 3).

Pearson’s bivariate correlation was used to analyze the relationship between age on admission and total inpatient hospital charges. Age at admission was positively correlated with total hospital charges in the primary OA of the left hip group (r = 0.05; p < 0.01) and primary right shoulder OA (r = 0.068; p < 0.05). Age at admission was negatively correlated with total hospital charges in bilateral knee OA (r = -0.115; p < 0.01), primary right knee OA (r = -0.054; p < 0.01), primary left knee OA (r = -0.060; p < 0.01), and unspecified primary knee OA (r = -0.036; p < 0.01) (Table 4).

**Table 4:** Single-layer mean comparison of the total charges and length of stay using 55 years as a cut-off, with bivariate correlation analysis of age (in years) at admission and total charges. ICD-10-CM, International Classification of Diseases, Tenth Revision, Clinical Modification; USD, U.S. dollars; SD, standard deviation; M1611, unilateral primary right hip osteoarthritis; M1612, unilateral primary left hip osteoarthritis; M170, bilateral primary knee osteoarthritis; M1711, unilateral primary right knee osteoarthritis; M1712, unilateral primary left knee osteoarthritis; M179, unspecified knee osteoarthritis; M19011, primary right shoulder osteoarthritis. ^1^Pearson’s bivariate correlation between age at admission and total hospital charges for each sub-group. *Correlation significant at 0.05 level (two-tailed). **Correlation significant at 0.01 level (two-tailed).

ICD-10-CM	Mean total charges (USD) ± SD		Mean length of stay (days) ± SD		R^1^
	≥55 years	<55 years	≥55 years	<55 years	
M1611	47,281 ± 18,918	48,726 ± 21,539	2.61 ± 1.527	2.20 ± 1.463	0.011
M1612	47,936 ± 23,398	47,346 ± 17,670	2.75 ± 2.764	2.07 ± 0.981	0.050*
M170	61,572 ± 29,173	60,678 ± 19,274	3.19 ± 1.724	2.88 ± 1.003	-0.115**
M1711	44,714 ± 22,616	44,471 ± 14,777	2.67 ± 1.805	2.33 ± 0.936	-0.054**
M1712	63,975 ± 30,323	43,795 ± 7,708	2.62 ± 1.441	3.08 ± 4.925	-0.060**
M179	45,488 ± 19,007	48,289 ± 18,224	2.75 ± 1.593	2.55 ± 1.171	-0.036**
M19011	52,966 ± 26,869	48,524 ± 17,164	1.95 ± 1.251	1.40 ± 0.563	0.068*

A single-layer comparison of means analyzed the length of stay (days) and total hospital charges (USD) within each of the included ICD-10-CM sub-groups, using 55 years of age as a layered threshold. The mean length of stay (days) was higher in patients aged > 55 years compared with those aged < 55 years for both primary right hip (2.61) and left hip (2.75) OA, though total hospital charges were greater in patients aged < 55 years with right hip OA (USD 48,726) and in patients aged > 55 years with left hip OA (USD 47,936). In patients with primary right shoulder OA, both length of stay (1.95) and total hospital charges (USD 52,966) were greater in patients older than > 55 years. In patients with primary OA of the right knee, both length of stay (2.67) and total charges (USD 44,714) were greater in patients older than 55 years, whereas in patients with left knee OA, both length of stay (3.08) and total charges (USD 47,918) were greater in patients younger than 55 years (Table 4).

For each ICD-10-CM sub-group, disposition and sex were analyzed using descriptive statistics. Disposition was decrypted using CMS "UB-04" disposition codes, and sex was coded on a binary system, with female = 1 and male = 0, with a mean value of 0.5 or greater signifying female predominance. The mean ± SD of sex and disposition were reported for each sub-group. Patients discharged following primary OA of the right and left hip, respectively, were more commonly females (0.57 ± 0.495 and 0.55 ± 0.498), with a mean disposition code of 5.26 ± 12.728 and 5.50 ± 13.391. Disposition counts following primary OA of the right and left knee, respectively, showed female predominance (0.63 ± 0.484 and 0.62 ± 0.487), with a mean disposition code of 4.88 ± 11.768 and 4.95 ± 12.057. Similar results for primary bilateral OA of the knee, knee OA unspecified, and primary right shoulder OA were recorded (Table 5). In Table 5 each of the disposition codes is numerically coded using the "UB-04" coding [9]. For example, a mean value close to “2” means patients were more often discharged to a general hospital as inpatient; a mean close to “3” means patients were more often discharged to a skilled nursing facility; a mean close to “4” means patients were more often discharged to an intermediate care facility; a mean close to “5” means patients were more often discharged to another healthcare institution, unspecified; and a mean close to “6” means patients were more often discharged home under home health services.

**Table 5:** Hospital disposition and sex based on UB-04 standard CMS disposition codes. ICD-10-CM, International Classification of Diseases, Tenth Revision, Clinical Modification; M1611, unilateral primary right hip osteoarthritis; M1612, unilateral primary left hip osteoarthritis; M170, bilateral primary knee osteoarthritis; M1711, unilateral primary right knee osteoarthritis; M1712, unilateral primary left knee osteoarthritis; M179, unspecified knee osteoarthritis; M19011, primary right shoulder osteoarthritis ^1^Disposition codes are numerically coded [9]. ^2^The closer the mean is to “0” the greater the male predominance, whereas a mean closer to “1” represents female predominance.

ICD-10-CM	Disposition of patient (UB-04 standard coding)^1^			Indicator of sex^2^		
	N	Mean	Standard deviation	N	Mean	Standard deviation
M1611	10,198	5.26	12.728	10,192	0.57	0.495
M1612	8,651	5.50	13.391	8,651	0.55	0.498
M170	4,237	7.52	16.411	4,235	0.58	0.494
M1711	41,771	4.88	11.768	14,771	0.63	0.484
M1712	13,860	4.95	12.057	13,857	0.62	0.487
M179	6956	4.73	11.739	6,956	0.63	0.484
M19011	1411	2.30	6.141	1,410	0.54	0.498

## Discussion

Research aim

The purpose of this study was to characterize the prevalence of symptomatic OA in hospitals in a Midwestern state. The financial burden of symptomatic OA is expected to increase dramatically within the next two decades, and states with a greater obese, elderly, and at-risk population will experience disproportionately higher costs. The key findings of this study include confirmation of a higher prevalence of symptomatic OA in elderly, white Wisconsin females, with the total hospital charges being highest in bilateral knee OA. This study also found a weakly negative correlation between total hospital charges and age in patients with unilateral primary OA of the right knee, OA of the left knee, and unspecified knee OA. Thus, the key findings should promote targeted inpatient or pre-hospital intervention in patients with knee OA aimed at both preventing hospitalization and mitigating in-hospital cost expenditure depending on the stage of OA. Future studies are needed to characterize the cause of osteoarthritic admission (fracture, acute-on-chronic exacerbation), pre-hospital intervention (specialist or primary provider), and in-hospital course of action in order to highlight areas ripe for change. The clinical implications of this study stem from its utility in assessing various demographics and hospital outcomes from OA admissions to be used as an instrument for recognizing these patients in the office and hospital and focusing on hospital or practice-specific cost-reduction strategies.

Symptomatic osteoarthritis

OA is a chronic condition that when symptomatic causes pain and stiffness, commonly with evidence of radiographic joint disease. Symptomatic OA can be debilitating, and the healthcare costs incurred in managing symptomatic OA merit high-quality studies of its financial impact. This study highlights unilateral, primary OA of the right (24.58%) and left (23.07%) knees as the two most common admitting diagnoses of OA, though general knee OA exceeds 50% of admitting diagnoses when combined with bilateral knee OA and unspecified knee OA. Nationwide, the estimated average discounted lifetime cost of knee OA was USD 140,300, with direct medical cost accounting for around 129,000, and around 10% (USD 12,400) attributable to the conservative management of knee OA over 28 years [10]. Given the expected surge in the prevalence of OA as the American population ages concurrently with an obesity epidemic, states such as Wisconsin whose residents have a greater number of risk factors may contribute disproportionately to incurred hospital costs, especially from knee OA.

Age

One of the strongest risk factors for the progression of OA in any joint is age [11]. Patients admitted with primary OA of the right shoulder were the oldest (mean age: 69.62 years), followed by OA of the right and left hip (67.1 years), with the youngest OA admissions occurring in the knees: unspecified (66.94), right (66.84), left (66.65), and bilateral (64.46). An aged joint is more likely susceptible to biologic change as a result of cumulative exposure to a variety of risk factors. According to the Division of Public Health Bureau of Aging and Disability Resources, the Wisconsin Department of Administration predicts a 21-51% increase in residents aged 60 years or older by 2040 [12]. Though a non-modifiable risk factor, the aged joint may be mitigated by preventative efforts, novel pharmaceuticals, or bettering other modifiable risk factors such as obesity and diet, and Wisconsin’s projected population growth merits further investigation in this regard.

Gender and race/ethnicity

Two common non-modifiable risk factors are gender and race or ethnicity, and while women are not only at a greater risk for OA than men, they also have more severe OA [13]. Racial and ethnic contributions to OA have been well studied, and a review of the literature suggests that it varies by joint. In one study, African American subjects displayed greater pain sensitivity and temporal summation than NHW adults with knee OA, though differences in clinical pain were non-significant when controlled for education and household income [14]. Similarly, results of the Johnston County Osteoarthritis Project showed a similar prevalence of hip OA in African American women (23%) compared with white women (22%), with more advanced radiographic hip OA in African American women compared with white women in an aged-matched cohort [15,16]. However, according to the U.S. Census data from 2015-2018, five-year estimates suggest that 93.1% of the Wisconsin population 65 years or older is NHW, with only 3.1% African American, compared with national averages of 76.5% for NHW and 9.1% for African Americans. Additionally, the percent of Wisconsin females aged 65 years or older is 54.8%, and the percent of these females living alone (35.9%) is greater than the national average (32.1%) [17]. These data suggest a disproportionately higher NHW population compared with national averages, as well as a greater percentage of elderly Wisconsin females than males. Similarly, findings from this study show a higher population of Wisconsin NHW and female inpatients with OA, as well as more females being sent to intermediate care facilities following hospital admission, making this demographic ripe for preventative efforts and initiatives that may influence future cost savings.

Obesity and occupation

Obesity, defined as a BMI of 30.0 or higher according to the World Health Organization, has long been recognized as a potent modifiable risk factor of OA, particularly in the knee [10]. According to the Centers for Disease Control and Prevention (CDC), the National prevalence of self-reported obesity among NHW adults is 29.3%, though in Wisconsin, this percentage for NHW adults approaches 34.9% [4]. Furthermore, the Framingham study showed that women who lost five kg of weight had a reported 50% reduction in risk of symptomatic knee OA [18]. While this study did not include BMI as a variable in assessing the inpatient demographics of OA, there certainly may lie a benefit in state-funded diet and activity promotion given the higher-than-national average of obese Wisconsin NHW adults as well as females. Despite Wisconsin’s total inpatient OA hospital charges being the lowest in primary right knee OA (USD 44,689) and primary left knee OA (USD 44,427), the combined knee OA group (along with bilateral knee OA and unspecified knee OA) exceeded greater than half of all OA admissions, which certainly warrants further investigation given its sheer volume.

Occupation has long been identified as a modifiable risk factor for general OA, and studies have shown that farmers have a higher prevalence of hip OA than other joints, with certain manual laborers, in general, carrying a two times greater risk of knee OA than the general population [19,20]. This study found that on average, for primary OA of the right and left hip, mean inpatient hospital costs (USD) and lengths of stay (days) were greater than those for unilateral primary OA of the knee or right shoulder. In Wisconsin, one of the top 10 agricultural states according to the U.S. Department of Agriculture Economic Research Service, targeted intervention within the population of farmers or manual laborers may be of significant financial benefit [21].

The strengths of this study lie in the large database available for analysis. Additionally, the large volume of admissions secondary to OA allowed for a sufficient sample size to be used in statistical analysis. There exist a few limitations to this study. This was an epidemiological analysis, and as such the accessible variables are limited. In this study, the few factors not considered include pre-admission duration of disease, radiographic stage of OA at the time of admission, discharge trajectory, and previous medical and social history including BMI, co-morbid joint disease, or pre-admission level of function, which may alter the results significantly. The causal association between total hospital charges or length of stay and each the most common OA diagnoses cannot be drawn as this design was cross-sectional, and thus the association is unclear. From an admission standpoint, conditions such as knee OA may have been classified as unspecified knee OA or lacked specific laterality, when, in theory, a more specific ICD-10-CM code could have been assigned. Therefore, in the discussion, knee OA was considered holistic. Furthermore, only patients with admitting diagnoses of OA were included in this study, and therefore the true breadth of arthritis could have been underestimated if other inpatients with more significant primary admitting codes had underlying joint disease. Last, though most important, these data were unable to distinguish between re-hospitalizations, and an accurate estimate of the total inpatient burden cannot accurately be made due to the composition of the state administrative database. However, the primary strength of this study is the volume of included admissions and its homogenous dataset derived solely from Wisconsin admissions. Additionally, using patient inclusion criteria based on ICD-10-CM diagnosis codes, the influence of selection bias was reduced.

## Conclusions

Using the SID dataset, this study found that the prevalence rate of OA admissions was highest in primary right and left knee OA. Additionally, in Wisconsin admission records from 2016, total inpatient charges were highest in admission for bilateral knee OA and primary OA of the right and left hip. These findings, in addition to risk factors explored herein, provide clinically significant data for the early diagnosis and intervention in OA patients. Particular attention should be paid to elderly, female, and Caucasian patients with hip and knee OA, as early intervention could have a drastic impact on reduction in statewide healthcare utilization and hospital costs. From a comprehensive literature review, this is the first statewide inpatient study, including 153 Wisconsin hospitals, to classify demographics and various inpatient outcomes associated with OA, such as length of stay, total charges, disposition, and mortality. Principally, these findings should highlight the need for future preventative efforts required to mitigate the increase in OA healthcare expenditures that is imminent with our aging population and obesity epidemic.
